# In Vitro Analysis of Aerodynamic Properties and Co-Deposition of a Fixed-Dose Combination of Fluticasone Furoate, Umeclidinium Bromide, and Vilanterol Trifenatate

**DOI:** 10.3390/pharmaceutics16101334

**Published:** 2024-10-18

**Authors:** Kittipong Maneechotesuwan, Somchai Sawatdee, Teerapol Srichana

**Affiliations:** 1 Department of Medicine, Division of Respiratory Disease and Tuberculosis, Faculty of Medicine Siriraj Hospital, Mahidol University, Bangkok 10700, Thailand; kittipong.man@mahidol.ac.th; 2 Drug and Cosmetics Excellence Center and School of Pharmacy, Walailak University, Thasala, Nakhon Si Thammarat 80161, Thailand; somchai.sa@wu.ac.th; 3 Drug Delivery System Excellence Center, Faculty of Pharmaceutical Sciences, Prince of Songkla University, Hat Yai, Songkhla 90112, Thailand; 4 Department of Pharmaceutical Technology, Faculty of Pharmaceutical Sciences, Prince of Songkla University, Hat Yai, Songkhla 90112, Thailand

**Keywords:** co-deposition, fixed-dose combination drug, fluticasone furoate, lower stages, umeclidinium bromide, vilanterol trifenatate

## Abstract

Background/Objectives: Effective airway delivery of a fixed-dose combination of triple-aerosolized inhaled corticosteroid (ICS)/long-acting beta agonist (LABA)/long-acting muscarinic antagonist (LAMA) is likely to positively affect therapeutic responses predicted in patients with asthma and chronic obstructive pulmonary disease. This study aimed to conduct in vitro fluticasone furoate, vilanterol trifenatate, and umeclidinium bromide depositions in a Next Generation Impactor. The aerodynamic properties of these inhaled medications influence the spatial distribution and drug abundance, particularly in the smaller airways, to reverse or alleviate disease pathology. Methods: The Next Generation Impactor was used to demonstrate the aerodynamic particle size distributions of fluticasone furoate, vilanterol trifenatate, and umeclidinium bromide delivered from a dry powder inhaler at different flow rates across all stages of the impactors. This in vitro study analyzed the distribution pattern of individual drug components to simulate mono-component deposition and co-deposition in the official model in the United States Pharmacopeia. An Andersen cascade impactor together with scanning electron microscope–energy-dispersive X-ray was employed to observe the drug deposition on each stage of the impactor. Results: We found that the distribution pattern of each component at the same cascade level was comparable, and the aerosol particles of the three drugs reached the in vitro representation of the lower airway compartment. The specified flow rates generated the desired fine particle fraction, fine particle dose, and mass median aerodynamic diameter. Our results also demonstrated visualized deposition patterns of the delivered drugs from different stages of the cascade impactor that may predict deposition as it occurs in vivo. Conclusions: Spatial distribution and abundance of ICS/LABA/LAMA in the same cascade levels were closely comparable, and the aerosol particles were able to reach the small aerosol-sized cascades at the lower levels to some extent.

## 1. Introduction

Small airway engagement is a primary pathophysiological feature that symptomatically burdens patients with chronic obstructive pulmonary disease (COPD) and uncontrolled asthma [1]. Pathological small airways in both of these uncontrolled diseases are mediated through inflammatory and remodeling processes that result in abnormal airway narrowing with subsequent lung hyperinflation and enhanced airway hyperresponsiveness [2]. Targeting the distal airways with inhaled medication results in decreased airway narrowing and further reduces dynamic hyperinflation in COPD [1,3]. All data have suggested that co-deposition of inhaled anti-inflammatory agents and a long-acting bronchodilator may enhance the possibility of cumulatively reversing or alleviating persistent small airway abnormalities in asthma and COPD.

Fixed-dose combinations of aerosolized bronchodilators and steroids are currently recommended for the treatment of asthma and COPD. Single-inhaler triple therapy of an inhaled corticosteroid (ICS), long-acting beta agonist (LABA), and a long-acting muscarinic antagonist (LAMA) reduces future risks for asthma and COPD exacerbations and all-cause mortality in symptomatic COPD patients [4,5]. Triple therapy also improves lung function in patients with COPD to a greater extent than fixed-dose dual LAMA/LABA or ICS/LABA therapies [6]. Mechanistically, it may require co-deposition of LABA and LAMA to relax the airway smooth muscles and with an ICS to suppress inflammation along the affected airways [7,8]. To date, very few studies have demonstrated the extent of particle-size-dependent co-deposition of fixed-dose triple-drug regimen delivered from a single inhaler device.

The TRELEGY ELLIPTA™ dry powder inhaler (DPI) contains two foil blister strips of powder formulations [6]. One strip contains fluticasone furoate (FF) (100 μg per blister) (ICS), and the other strip contains umeclidinium bromide (UMEC) (LAMA) and vilanterol trifenatate (VI) (LABA) (UMEC/VI 62.5/25 μg per blister). The inactive ingredients include lactose monohydrate and magnesium stearate [9]. In vitro drug deposition in cascade impactors is generally used for lung deposition prediction based on an in vitro–in vivo correlation [10]. The correlation between in vitro results and in vivo performance is dependent on various parameters associated with the in vitro experiment design. It consists of modeling of the upper airways, inhalation maneuvers, the orientation and positioning of the inhaler in relation to the mouth, and the expected environmental variables such as temperature, humidity, and ambient pressure [11]. The in vitro deposition by cascade impactor analysis of a DPI is a standard procedure that is officially documented in the United States Pharmacopeia (USP) [12]. However, it is important to note that the data from in vivo results must be carefully interpreted. Guidance for good cascade impactor practice is presented in the USP and in the literature [10,13,14].

The present study aimed to demonstrate the degree of fixed-dose FF/UMEC/VI co-deposition using an in vitro model of several cascades to mimic drug delivery via the DPI. We also examined the configuration of the drug particle distribution in the cascade impactor based on a prior determination of the aerodynamics to generate optimal particle size in response to various flow rates. Furthermore, co-deposition was visualized by scanning electron microscopy (SEM) and the energy-dispersive X-ray (EDX) technique.

## 2. Materials and Methods

### 2.1. Materials

TRELEGY ELLIPTA™ (DPI) devices were gifted from GlaxoSmithKline, Thailand. The individual components of the TRELEGY ELLIPTA™ (DPI) (Lot no. VT5H, GlaxoSmithKline UK Ltd., Durham, UK), which contained FF, UMEC, and VI at doses of 100 μg, 62.5 μg, and 25 μg, respectively, were investigated to observe aerodynamic particle size deposition after emission from the tested DPI.

### 2.2. Life-Mimicking Testing System

The life-mimicking testing system was undertaken over a period of 30 days to determine the impact of simulated environmental stress (dropping, vibration, and moisture) on delivered dose (DD) consistency of the in vitro dose delivery. Two batches of Trelegy were used to assess dose delivery performance over the inhaler lifespan (doses 1–3, doses 15–18, doses 28–30). Fine particle dose (FPD) and the delivered doses were evaluated. Delivered doses were dispensed for 4 s using a unit dose collector (Copley Scientific Limited, Nottingham, UK) at a flow rate of 60 L/min, and the retrieved doses were examined in triplicate experiments.

### 2.3. Delivered Dose Against Different Flow Rates

**Figure 1:**
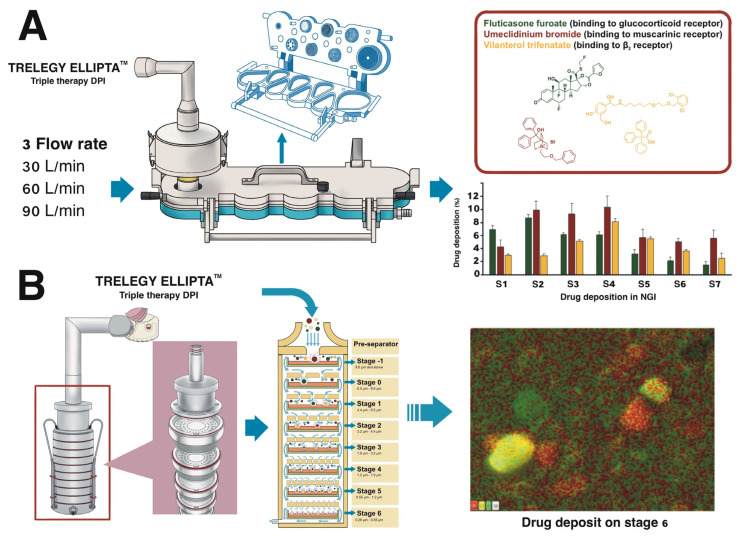
(**A**) The schematic diagram represents the experimental design for drug deposition from a TRELEGE ELLIPTA™ DPI inhaler (fluticasone furoate, umeclidinium bromide, vilanterol trifenatate) in a Next Generation Impactor (NGI) at different air flow rates. (**B**) The experimental diagram of drug deposition in an Andersen cascade impactor (ACI) to determine the distribution patterns at each stage using scanning electron microscopy (SEM) and an energy-dispersive X-ray (EDX) analysis system. The drugs are presented by different colors in the bar chart and SEM images. The green color is fluticasone furoate, the red color is umeclidinium bromide, and the yellow/orange color is vilanterol trifenatate.

Delivered dose was performed according to the test for delivered dose uniformity under the guidance for metered-dose inhalers and dry powder inhalers [12]. The two-strip configuration of the DPI was tested using two doses from the DPI (100/62.5/25 µg) stratified throughout the 30-dose inhaler. The dose delivery performance was ascertained by a Next Generation Impactor (NGI). Measurements were conducted on the FPD (i.e., mass of particles less than 5 µm) of each mono-component against the flow rates of 30, 60, and 90 L/min throughout the inhalation volume of 4 L in the NGI. The three active components were analyzed by high-performance liquid chromatography (HPLC) on six consecutive occasions. The experimental diagram of this section is shown in Figure 1A.

### 2.4. Delivered Dose Uniformity

The uniformity of the DD was evaluated according to the USP with a minor modification to determine the content of the active substances delivered from the mouthpiece of the inhaler device [12]. Briefly, the DPI was horizontally connected to the sampling apparatus B as described in the USP with a mouthpiece adapter [12]. We adjusted the apparatus to obtain an air flow rate of 60 L/min, followed by discharging one dose of the powder into the sampling apparatus. The DPI was then detached from the sampling apparatus, and the vacuum tubing was disconnected. The filter and interior of the sample collection tube were rinsed with the mobile phase and analyzed by HPLC. This procedure was repeated for each of the 10 separate unit doses to determine the uniformity.

### 2.5. Microscopic and Elemental Analysis

The eight-stage Andersen cascade impactor (ACI) was used to characterize the deposition of each drug component at 60 L/min for 4 s. Determination of the distribution patterns at each stage was conducted using scanning electron microscopy (SEM) and an energy-dispersive X-ray (EDX) analysis system. Double adhesive discs (3M, Bangkok, Thailand) were prepared and placed on each collection plate to trap the particles that lost momentum. The ACI was operated at 60 L/min for 4 s. The drug deposited on each stage of the ACI was collected for observation under SEM–EDX. The SEM–EDX analysis of each stage of the impactor (stages −1, 0, 1, 2, 3, 4, 5, and 6) was conducted for each drug component. The three drugs, FF, UMEC, and VI, were mapped in green, red, and yellow, respectively, from the EDX analysis. The experimental diagram of this section is also shown in Figure 1B.

### 2.6. High-Performance Liquid Chromatography (HPLC)

An HPLC machine (Thermo Scientific, Waltham, MA, USA) was employed in the present investigation. The machine included a SpectraSYSTEM™ SCM1000 degasser, SpectraSYSTEM P2000 Pump, SpectraSYSTEM AS3000 autosampler, SpectraSYSTEM UV1000 detector, and an SN4000 program controller operated by ChromQuest software version 5.0. Separation was attained by a C18 stationary phase column (BDS 150 × 4.6 mm, 5 μm) (Thermo Fischer Scientific, Winsford, UK). The mobile phase contained a combination of 0.6% (*w*/*v*) ammonium acetate in water, methanol, and acetonitrile (40:40:20 *v*/*v*, respectively). A flow rate of 1 mL/min at ambient temperature and injection volume of 50 μL were used in the mobile phase. Detection was conducted at a wavelength of 228 nm. The analytical validation including accuracy, linearity, and inter- and intra-day precision were performed before analysis.

### 2.7. Statistical Analysis

All experiments were performed independently five times. Data are expressed as mean ± standard deviation. The Shapiro–Wilk test and Levene’s test were performed to confirm the distribution and analyze the variance before the statistical analysis. The statistical analysis was carried out using Python 3.0 (Python Software Foundation, Amsterdam, The Netherlands). Comparisons were performed using the Tukey test. Statistical significance was defined as *p* < 0.05.

## 3. Results

### 3.1. Analysis of FF/UMEC/VI by HPLC

**Figure 2:**
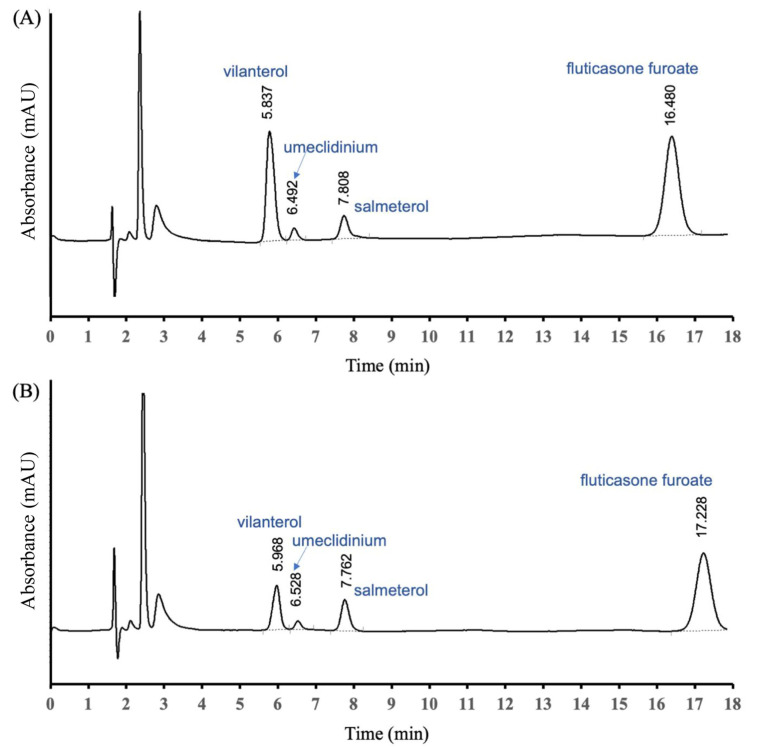
Chromatogram representing the peaks and retention times of (**A**) standards vilanterol, umeclidinium bromide, salmeterol, and fluticasone furoate and (**B**) sample of vilanterol, umeclidinium bromide, and fluticasone furoate from a TRELEGE ELLIPTA™ DPI inhaler with the spike of the salmeterol internal standard.

Figure 2 shows the chromatographic system for analysis of FF/UMEC/VI with standards and salmeterol as an internal standard. The results indicated that the analysis system was accurate and precise with 98–101% for VI, 97–99% for UMEC, and 98–101% for FF recovery, and the percent coefficient of variation was 1.1–1.8. The linearity over 2–12 µg/mL of all three drugs was 0.9985–0.9995, which was within the acceptable range. The retention times of VI, UMEC, FF, and salmeterol were 5.8, 6.5, 7.8, and 16.5 min, respectively. The chromatograms of the standards were similar to the samples (Figure 2).

The contents of the TRELEGE ELLIPTA™ DPI were VI 116.21% ± 7.82%, UMEC 97.75% ± 6.06, and FF 97.10% ± 1.39%. The respective emitted doses of VI, UMEC, and FF were 95.31% ± 4.97%, 82.99% ± 3.01%, and 74.96% ± 4.88%. The fine particle fraction (FPF) values of the three active ingredients were 57.75 ± 0.78%, 59.19 ± 0.53%, and 42.91 ± 2.19% for VI, UMEC, and FF, respectively. The mass median aerodynamic diameter (MMAD) values were 2.32 μm ± 0.11, 2.20 μm ± 0.02, and 2.34 μm ± 0.03 for VI, UMEC, and FF, respectively. Polydisperse systems were observed in all three drugs, and the geometric standard deviation (GSD) values were 1.88 ± 0.02, 1.96 ± 0.01, and 1.73 ± 0.04 for VI, UMEC, and FF, respectively.

### 3.2. FF/UMEC/VI Delivered from the Ellipta™ DPI Were Fine Particles and In Vitro Could Reach the Small Airway Stages

Table 1 shows the deposition percentages of FF, UMEC, and VI at 30, 60, and 90 L/min in the NGI stages 1–7. Depositions of FF, UMEC, and VI were high in both the metal inlet (15–25% or 15–25 μg, 6–15% or 4–9 μg, and 7–15% or 2–4 μg, respectively) and preseparator (41–42% or 41–42 μg, 32–40% or 20–26 μg, and 56–58% or 14–15 μg, respectively) (Table 2). Therefore, lower depositions were observed in stages 1–7 of the impactor. When the data were analyzed for normal distribution, all *p*-values were >0.05, which indicated a normal distribution with equal variance (Table 1). Most of the FF was deposited on stages 1–4, and a smaller amount was deposited on stages 5–7. However, UMEC deposited mainly on stages 2–5, and a higher deposition percentage (4–5%) was also found on stages 6–7 at the higher flow rates (60 L/min and 90 L/min). Higher flow rates yielded higher depositions on the lower stages of the NGI. VI deposition was found to be high on stages 3–5 and at the higher flow rates of 60 L/min and 90 L/min, with higher deposition percentages on stages 6 and 7.

**Figure 3:**
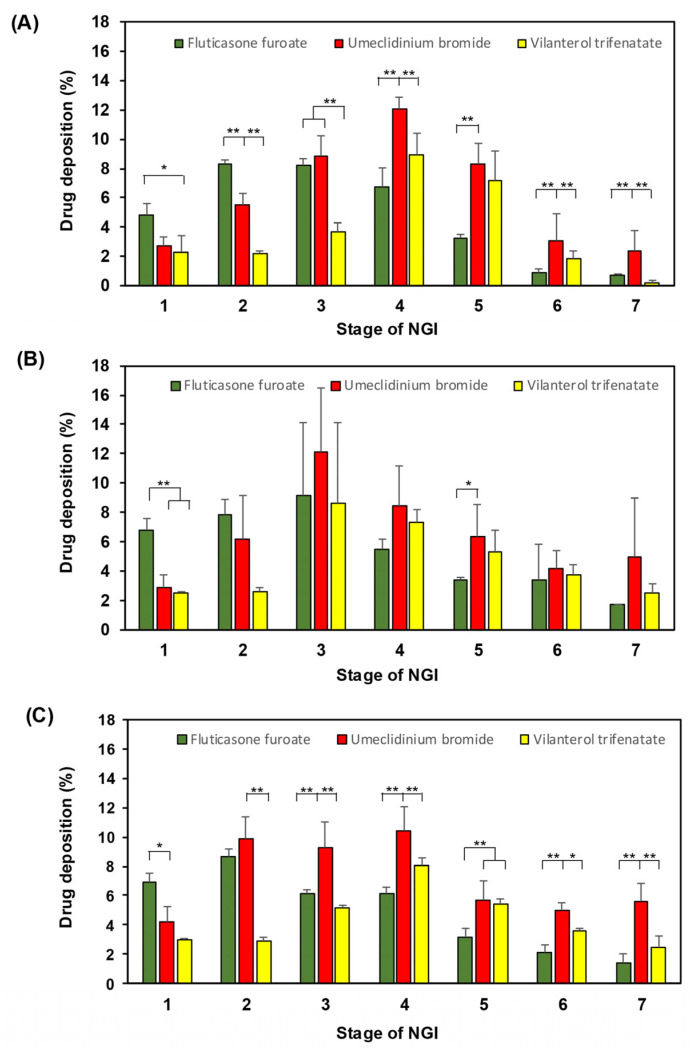
Aerodynamic particle size distributions of vilanterol trifenatate (green bar), umeclidinium bromide (red bar), and fluticasone furoate (yellow bar) from the TRELEGE ELLIPTA™ DPI inhaler operated with the Next Generation Impactor (NGI; USP Apparatus 6) at different air flow rates of (**A**) 30 L/min, (**B**) 60 L/min, and (**C**) 90 L/min. Significance by Tukey test was defined as *p*-value < 0.05 (*) or *p*-value < 0.01 (**).

Figure 3 compares the drug depositions of different drugs on different stages at given flow rates. At 30 L/min, the deposition percentages of FF and UMEC were different except on stage 3 (Figure 3A). However, UMEC and VI had similar deposition percentages only on stages 1 and 5, whereas all drugs were significantly different on the other stages (*p* < 0.01). At the higher flow rate of 60 L/min, statistical differences were found on stages 1 and 5, where FF was the highest on stage 1 and the lowest on stage 5 compared to UMEC and VI (Figure 3B). There were no differences on the other stages (2, 3, 4, 6, and 7) from any drug. At the highest flow rate of 90 L/min, FF deposition was different from UMEC and VI on stages 1–7 except stage 2 (Figure 3C). The FPF was found to be the highest at the flow rate of 60 L/min and the lowest at 90 L/min for FF (Table 3).

At higher flow rates, higher deposition was due to particle loss from inertial impaction. For example, the FPF increased from 30 L/min to 60 L/min and then decreased from 60 L/min to 90 L/min in the case of FF. UMEC increased from 35% (22 μg) to 40% (25 μg) at flow rates from 30 L/min to 60 L/min, then slightly decreased from 60 L/min to 90 L/min. Deposition for VI followed a similar trend that went from 22% (5 μg) to 30% (7 μg) and then slightly decreased at 90 L/min. The overall results of the NGI deposition at different flow rates for FF/UMEC/VI with in vitro aerosol performance characteristics (DD, FPF, FPD, MMAD, and GSD) are shown in Table 3. The FPF values were similar for the ICS (FF) and LABA (VI) components, which were 20.34% and 22.10% at 30 L/min and 29.35% and 30.43% at 60 L/min, respectively. At 90 L/min, the FPF value decreased from 30% to 20% in the case of FF. However, the FPF values of the LAMA (UMEC) component were 35.64 at 30 L/min and 41.62 at 60 L/min. The MMAD values for FF/UMEC/VI were similar across the three components (2.37–3.12 μm at 30 L/min and 1.62–2.18 μm at 60 L/min). The GSD values were 1.58–2.49 at 30 L/min and 1.49–2.53 at 60 L/min across the three components. An analysis of the individual components using the NGI showed a significant difference in the FPF, MMAD, and GSD values across the different flow rates. There was little evidence of trends in the FPD for each component across the flow rate profiles tested. The FPD values for UMEC and VI were similar except for VI at 60 L/min, which was relatively higher than the flow rates of 30 and 90 L/min, while the FPD of FF was slightly higher than either UMEC or VI.

**Figure 4:**
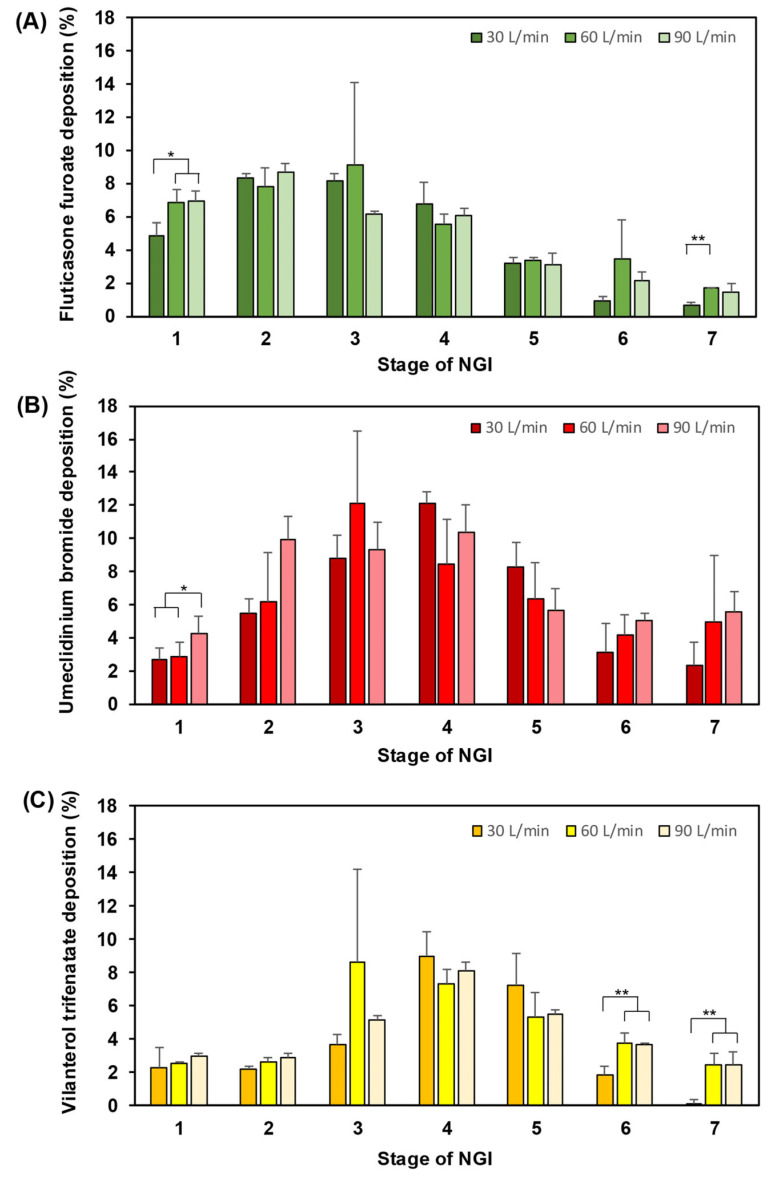
Aerodynamic particle size distributions: (**A**) vilanterol trifenatate; (**B**) umeclidinium bromide; and (**C**) fluticasone furoate. The TRELEGE ELLIPTA™ DPI inhaler was operated with the Next Generation Impactor (NGI; USP Apparatus 6) at different air flow rates of 30, 60, and 90 L/min. Significance by Tukey test was defined as *p*-value < 0.05 (*) or *p*-value < 0.01 (**).

Figure 4 shows FF/UMEC/VI depositions compared at different flow rates. The depositions of FF were not different at the three flow rates except on stages 1 and 7 at 30 L/min (Figure 4A). Significant differences were noted on stage 1 at 30 L/min and 60 L/min and on stage 7 at 30 L/min (*p* < 0.05). The deposition of UMEC on stage 2 (Figure 4B) was not different at any flow rate, which indicated that UMEC was not flow-sensitive except at stage 1. Furthermore, VI deposition also was not different on stages 1–5 at any flow rate (Figure 4C). Only stages 6–7 of VI were different at 30 L/min, 60 L/min, and 90 L/min (*p* < 0.01).

The deposition of aerodynamic particles at stage 3 and stage 4 of the NGI represents the regional small airways which have cut-off diameters less than 3.99–1.31 μm depending on the air flow rate. On the other hand, differences in the representative regional small airway deposition across the flow rate profiles for UMEC were not seen in stage 6 or stage 7 (Figure 4B). The mean representative regional small airway deposition for VI as a percentage of DD was significantly higher at 60 L/min than at 30 L/min in stage 3 (Figure 4C).

### 3.3. Delivered Doses of FF/UMEC/VI from the Ellipta™ DPI Mimicked the Testing System over a Period of 30 Days

The mean ± SD percentages of the DDs of FF, UMEC, and VI were 72.0 ± 2.5, 82.3 ± 3.5, and 94.9 ± 5.6, respectively, across the flow rate profiles. In stages 3–5 of the NGI, which represented small airway deposition, the percentages of FF, UMEC, and VI were 15–18% (equivalent to mass 15–18 μg), 26–29% (equivalent to mass 16–18 μg), and 18–21% (equivalent to mass 4–5 μg), respectively, across the flow rate profiles. The mean central NGI deposition (stage 2) as a percentage of the DD for each component was lower than the representative regional small airway cascades. Differences in the representatives of the NGI lower-stage depositions (stages 3 and 4) for FF at 30 L/min versus 60 L/min were all statistically significant (nominal *p* < 0.05). The DD values from the two batches remained consistent throughout the inhaler lifespan, and the simulated environmental stress did not affect its performance (% relative standard deviation < 3.5). Similar DD values were observed with or without dropping, vibration, or exposure to moisture.

### 3.4. SEM Images Demonstrated Co-Deposition of Triple-Drug Aerosols in Each Stage of the ACI

**Figure 5:**
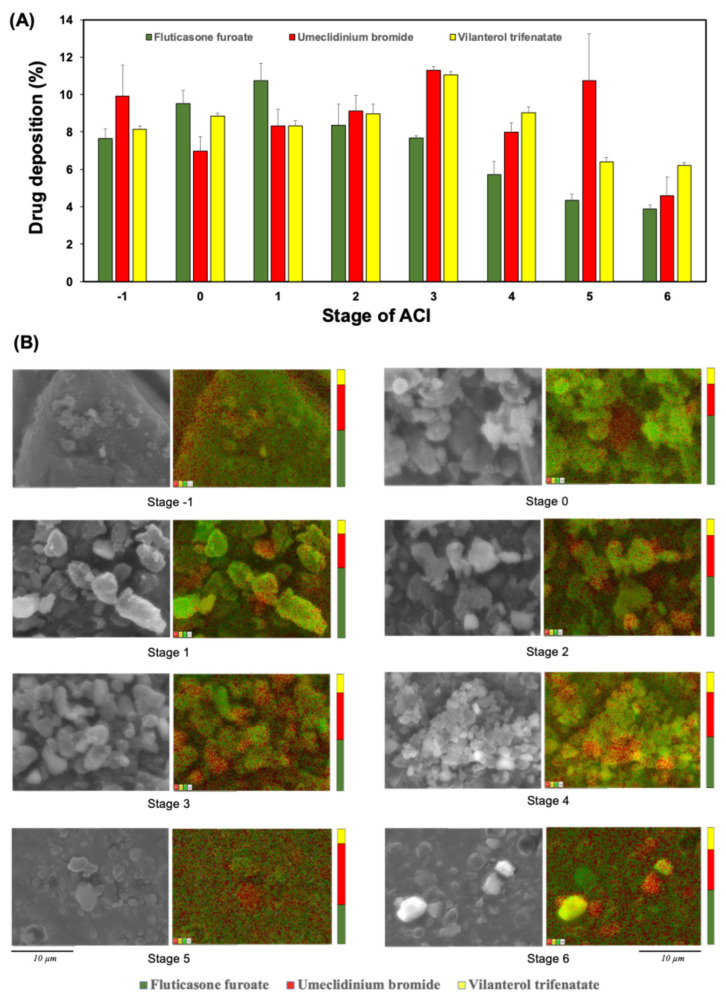
Aerodynamic particle size distributions of vilanterol trifenatate (green bar), umeclidinium bromide (red bar), and fluticasone furoate (yellow bar) from the TRELEGE ELLIPTA™ DPI inhaler obtained with the Andersen cascade impactor (ACI; USP Apparatus 1). (**A**) Scanning electron microscopy (SEM) images of the fixed-dose combination of ICS/LABA/LAMA particle co-deposition on each stage of the ACI under SEM and energy-dispersive X-ray (EDX) qualitative analyses. Red represents the Br atom of umeclidinium bromide, yellow represents the Cl atom of vilanterol trifenatate, and green represents the F atom of fluticasone furoate. (**B**) The bars indicate the proportions of drug deposition at each stage of the ACI by normalized EDX signal analysis.

Figure 5 shows a representative visualization of the co-deposition patterns of each component of FF/UMEC/VI tagged with their respective immunofluorescence. The SEM images revealed that all drugs had a uniform distribution, and an overlay of their immunofluorescence signals was observed in the lower stages of the impactor. Although UMEC provided the predominant immunofluorescent signal over the remaining drugs throughout all stages of the impactor, the immunofluorescent intensities of FF and VI were similar. This suggested that a fixed-dose combination of aerosolized VI, UMEC, and FF was effectively delivered to the same area in in vitro compartments. Early deposition was found on stages −1, 0, 1, and 2 in all three drugs (Table 4). FF was lower on stages 3–6 than UMEC and VI, which demonstrated that UMEC/VI can go deeper into the lungs. The deposited masses of FF, UMEC, and VI found on the lower stages (stages 3–6) of the ACI were 21.59 ± 1.70 μg, 21.61 ± 1.93 μg, and 8.18 ± 0.58 μg, respectively. It was concluded that about half of the active drug was deposited on stages 0–2 (Table 4).

## 4. Discussion

We demonstrated that the aerodynamic attributes of aerosolized VI, UMEC, and FF delivered from the Ellipta™ DPI bore resemblance leading to a comparable spatial distribution and effective small airway cascade deposition. One way to interpret and apply the current deposition data in a clinical context is to match the data with the distribution of the corresponding receptors along the human airway and their possible synergism. Glucocorticoid receptor expression and binding in human airways matched our deposition data for an ICS since FF was abundant across all stages, which implied anti-inflammatory effectiveness of the ICS in controlling large and small airway inflammation [15,16]. This was the case for the spatial distribution of VI and UMEC in the context of the mean deposition of both aerosolized drugs in the early and further impactor stages. Furthermore, no disparity was shown, and this profile may compensate for the relatively different abundances of M3-receptors and β_2_-receptors along human airways, which may possibly enable simultaneous binding to M3-receptors and β_2_-receptors [7]. The uniform aerodynamic particle size distribution of all aerosolized drugs in our results may explain the clinical effectiveness of a single-inhaler triple therapy in COPD despite a stark contrast with a previous study of a fixed combination of ICS/LABA [8].

The co-deposition of fixed-dose combination triple-aerosolized drugs at in vitro small airway levels shown in our study reflects the importance of optimal size distribution and likely increases the possibility of their own receptor binding with subsequent synergistic effects [8]. Our data also support previous findings that addressed the co-deposition of ICS and LABA particles in a combination metered-dose inhaler that was significantly greater than with the use of separate inhaler devices [17]. The significance of co-deposition was highlighted in the context of supra-additive and synergistic effects by mechanistic studies of the positive interaction between ICS and LABA, LAMA and LABA, and ICS and LAMA [18,19,20]. The aerodynamic characteristics of the fixed-dose combination of FF/VI/UMEC in the TRELEGY ELLIPTA™ may lead to improvement in small airway deposition that translates into clinical efficacy for delivery of both inhaled corticosteroids and LABA as previously demonstrated [21]. However, some limitations may be noted in the present study. This was an in vitro study that may not fully describe in vivo airway deposition of triple-aerosolized drugs that occurs in asthmatic and COPD patients, which is complicated by abnormal airway geometry and temporal and spatial relationships between the large and small airways. Also, the cascade impactor may give better predictive data if equipped with an Alberta Idealized Throat, which is an improved design of a metal inlet [11].

In addition, the current study could not determine pharmacokinetic and pharmacodynamic correlations among the different aerosolized drugs. Therefore, further studies are required to gain a better understanding of this aspect, including investigations of the synergistic effects in patients with asthma and COPD. Finally, a hypothetical test of whether a higher degree of co-deposition on the same cells of the airways may possibly account for the increased efficacy observed in asthmatic and COPD patients is clearly warranted.

When considering the FF/UMEC/VI particle characteristics, a lower FPF was observed with an unchanged MMAD for the ICS component compared with the LAMA component but not with the LABA component across the flow rate profiles as previously reported by three-dimensional airway models [22]. The FPD of the FF, UMEC, and VI components across the flow rate profiles in the current study was different from previous reports that demonstrated little flow dependency [23]. Our work demonstrated that the flow rate had a strong impact on the FPD. The FPD increased with increased flow rate from 30 L/min to 60 L/min due to de-aggregation of the particles at the higher flow rate. However, increased flow rate also increased inertial impaction. In the case of 90 L/min, the deposition loss was from impaction that resulted in a significant decrease in FPD, although the MMAD likely decreased. Based on a previous study of in vitro performance of the ELLIPTA™ DPI, we assumed that the FPD of the FF and UMEC components was consistent whether the component was delivered as a combination therapy or as monotherapy. Furthermore, the FPD of VI was also unaffected [23]. Therefore, the FPD possibly did not affect the co-deposition of FF/UMEC/VI in our study.

The DD results and SEM–EDX analysis in the current study showed that the ELLIPTA™ and the dry powder formulation uniformly delivered the drug component to the lower airways. In contrast to our data, a previous study showed that the in vitro performance of the ELLIPTA™ at an inspiratory flow rate of 41.6–136.9 L/min was consistent with delivering a combined drug compared to monotherapy [23]. The ELLIPTA™ showed a significant decrease in the FPF of all three drugs at 90 L/min, which was different from previous results obtained from a study using the Diskus^®^ DPI within the flow range of 60–90 L/min [24]. This may be due to the different designs of inhaler devices. A higher flow rate does not guarantee that higher drug deposition will be obtained, because higher inertial impaction may result in lost inertia of the drug particles at an early stage. Additionally, a patient’s capacity to produce enough flow for efficient DPI use is constrained and controlled by inspiratory pressure rather than flow rates [25].

## 5. Conclusions

This was an in vitro analysis of aerosol performance and co-deposition of FF/UMEC/VI delivered from a single inhaler device into an eight-stage ACI. The NGI demonstrated uniform co-deposition patterns of the delivered drugs from one to another at the representative small airway cascade. The findings showed that spatial distribution and the abundance of the ICS, LABA, and LAMA in the same cascade levels were closely comparable. Furthermore, the aerosol particles could reach the small aerosol-sized cascades at the lower levels to some extent, possibly increasing the possibility of drug co-deposition. The current study adds to the growing body of knowledge on small airway deposition of a triple fixed-dose combination that is a critical factor for management of uncontrolled asthma and COPD.

## Figures and Tables

**Table 1:** Deposition percentages of fluticasone furoate, umeclidinium bromide, and vilanterol trifenatate on the stages of NGI at different flow rates (mean ± SD, *n* = 5) and *p*-values from the Shapiro–Wilk test.

Flow Rate (L/min)	Stage of NGI	Fluticasone Furoate	*p*-Values	Umeclidinium Bromide	*p*-Values	Vilanterol Trifenatate	*p*-Values
	1	4.81 ± 0.80	0.408	2.69 ± 0.68	0.796	2.29 ± 1.17	0.441
	2	8.32 ± 0.30	0.996	5.51 ± 0.81	0.444	2.17 ± 0.22	0.083
	3	8.19 ± 0.45	0.408	8.82 ± 1.42	0.796	3.66 ± 0.59	0.441
	4	6.77 ± 1.32	0.372	12.12 ± 0.71	0.138	8.96 ± 1.44	0.133
30	5	3.20 ± 0.33	0.895	8.28 ± 1.47	0.703	7.19 ± 1. 96	0.125
	6	0.88 ± 0.29	0.851	3.09 ± 1.81	0.770	1.83 ± 0.53	0.744
	7	0.67 ± 0.14	0.061	2.35 ± 1.42	0.703	0.14 ± 0.18	0.743
	1	6.83 ± 0.78	0.693	2.86 ± 0.92	0.181	2.50 ± 0.14	0.961
	2	7.84 ± 1.09	0.730	6.14 ± 3.02	0.569	2.62 ± 0.27	0.473
	3	9.13 ± 5.00	0.298	12.13 ± 4.38	0.422	8.60 ± 5.56	0.235
	4	5.51 ± 0.69	0.446	8.48 ± 2.68	0.123	7.33 ± 0.84	0.333
60	5	3.37 ± 0.19	0.757	6.32 ± 2.19	0.311	5.34 ± 1.46	0.559
	6	3.43 ± 2.39	0.135	4.13 ± 1.24	0.716	3.78 ± 0.62	0.706
	7	1.71 ± 0.03	1.00	4.99 ± 3.95	0.867	2.48 ± 0.62	0.336
	1	6.92 ± 0.62	0.693	4.24 ± 1.04	0.398	2.96 ± 0.14	0.684
	2	8.66 ± 0.53	0.271	9.91 ± 1.42	0.146	2.88 ± 0.28	0.701
	3	6.14 ± 0.22	0.741	9.30 ± 1.68	0.312	5.16 ± 0.22	0.443
	4	6.10 ± 0.44	0.718	10.37 ± 1.67	0.917	8.07 ± 0.52	0.129
90	5	3.12 ± 0.67	0.704	5.68 ± 1.29	0.473	5.47 ± 0.27	0.529
	6	2.12 ± 0.56	0.222	5.01 ± 0.47	0.639	3.63 ± 0.14	0.523
	7	1.45 ± 0.54	0.233	5.58 ± 1.23	0.527	2.48 ± 0.77	0.791

The % deposition was calculated from the labelling of fluticasone furoate 100 μg, umeclidinium bromide 62.5 μg, and vilanterol trifenatate 25 μg. NGI: Next Generation Impactor.

**Table 2:** Masses and deposition percentages of fluticasone furoate, umeclidinium bromide, and vilanterol trifenatate on all NGI stages at three different flow rates (mean ± SD, *n* = 5).

Flow Rate (L/min)	Stage of NGI	Mass of Fluticasone µg %		Mass of Umeclidinium µg %		Mass of Vilanterol µg %	
30	Metal inlet	24.52 ± 4.26	24.52 ± 4.26	9.48 ± 1.98	15.16 ± 3.16	3.76 ± 0.38	15.04 ± 1.52
	Preseparator	42.02 ± 2.82	42.02 ± 2.82	25.62 ± 2.16	40.99 ± 3.46	14.60 ± 0.86	58.40 ± 3.43
	Stages 1–7	32.85 ± 2.02	32.85 ± 2.02	26.79 ± 1.90	42.86 ± 3.04	6.57 ± 0.66	26.26 ± 2.64
	Micro-orifice collector	0.62 ± 0.63	0.62 ± 0.63	0.62 ± 0.41	0.99 ± 0.66	0.11 ± 0.18	0.42 ± 0.73
	FPF < 2 µm	5.38 ± 0.42	5.38 ± 0.42	9.19 ± 1.50	14.71 ± 2.40	2.37 ± 0.57	9.48 ± 2.29
60	Metal inlet	15.77 ± 9.05	15.77 ± 9.05	4.03 ± 3.30	6.44 ± 4.84	1.89 ± 1.59	7.56 ± 6.36
	Preseparator	41.32 ± 5.41	41.32 ± 5.41	19.55 ± 5.73	31.28 ± 9.17	14.45 ± 0.81	57.78 ± 3.24
	Stages 1–7	41.18 ± 4.47	41.18 ± 4.47	35.42 ± 6.17	56.67 ± 9.87	8.10 ± 0.65	32.40 ± 2.58
	Micro-orifice collector	1.72 ± 0.44	1.72 ± 0.44	3.51 ± 1.68	5.62 ± 2.69	0.57 ± 0.19	2.26 ± 0.74
	FPF < 2 µm	17.95 ± 3.37	17.95 ± 3.37	23.04 ± 1.60	36.87 ± 2.56	6.03 ± 0.80	24.11 ± 3.20
90	Metal inlet	23.09 ± 0.87	23.09 ± 0.87	7.47 ± 0.48	11.95 ± 0.76	2.78 ± 0.04	11.13 ± 0.17
	Preseparator	41.30 ± 2.70	41.30 ± 2.70	20.61 ± 1.81	32.98 ± 2.90	14.15 ± 0.30	56.61 ± 1.19
	Stages 1–7	34.51 ± 2.63	34.51 ± 2.63	31.31 ± 1.99	50.10 ± 3.18	7.66 ± 0.23	30.64 ± 0.90
	Micro-orifice collector	1.10 ± 0.5	1.10 ± 0.5	3.11 ± 0.42	4.98 ± 0.67	0.41 ± 0.06	1.62 ± 0.23
	FPF < 2 µm	13.89 ± 2.32	13.89 ± 2.32	19.76 ± 1.20	31.62 ± 1.92	5.32 ± 0.39	21.27 ± 1.56

The % deposition was calculated from the labelling of fluticasone furoate 100 μg, umeclidinium bromide 62.5 μg, and vilanterol trifenatate 25 μg. NGI: Next Generation Impactor; FPF: fine particle fraction.

**Table 3:** DD, FPF, FPD, MMAD, and GSD values of fluticasone furoate, umeclidinium bromide, and vilanterol trifenatate at flow rates of 30, 60, and 90 L/min (mean ± SD, *n* = 5).

Drug	Flow Rate (L/min)	DD (%)	FPF (%)	*p*-Values	FPD (µg)	MMAD (µm)	*p*-Values	GSD
Fluticasone furoate	30	71.88 ± 2.53	20.34 ± 1.36	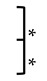	20.3 ± 1.4	3.12 ± 0.00	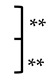	1.58 ± 0.02
	60	69.05 ± 7.74	29.35 ± 6.85		29.4 ± 6.9	2.18 ± 0.03		1.49 ± 0.20
	90	71.00 ± 2.09	20.03 ± 2.53		20.0 ± 2.5	1.77 ± 0.01		1.73 ± 0.05
Umeclidinium bromide	30	88.14 ± 2.53	35.64 ± 2.68	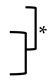	22.1 ± 1.6	2.37 ± 0.08	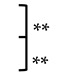	2.28 ± 0.05
	60	82.99 ± 3.01	41.62 ± 7.83		25.9 ± 4.9	1.75 ± 0.22		1.91 ± 0.54
	90	75.77 ± 4.54	40.93 ± 3.30		25.6 ± 2.1	1.22 ± 0.03		1.89 ± 0.13
Vilanterol trifenatate	30	99.46 ± 3.77	22.10 ± 3.60	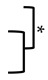	5.5 ± 0.9	2.37 ± 0.05	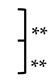	2.49 ± 0.52
	60	95.31 ± 3.28	30.43 ± 4.80		7.6 ± 1.2	1.62 ± 0.10		2.53 ± 0.15
	90	90.27 ± 2.45	26.42 ± 1.38		6.6 ± 0.3	1.22 ± 0.02		2.29 ± 0.11

* The FPF was calculated from the % labeled claim of fluticasone furoate 100 μg, umeclidinium bromide 62.5 μg, and vilanterol trifenatate 25 μg. DD: delivered dose; FPF: fine particle fraction; FPD: fine particle dose; MMAD: mass median aerodynamic diameter; GSD: geometric standard deviation. The asterisk (*) indicates the significant differences from Tukey test; * *p*-value < 0.05; ** *p*-value < 0.01.

**Table 4:** Masses and deposition percentages of fluticasone furoate, umeclidinium bromide, and vilanterol trifenatate on each stage of the Andersen cascade impactor (ACI) at flow rates of 60 L/min (mean ± SD, *n* = 3).

Stage of ACI	Mass of Fluticasone		Mass of Umeclidinium		Mass of Vilanterol	
	μg	%	μg	%	μg	%
−1	7.66 ± 0.50	7.66 ± 0.50	6.19 ± 1.04	9.91 ± 1.67	2.04 ± 0.05	8.15 ± 0.18
0	9.52 ± 0.70	9.52 ± 0.70	4.35 ± 0.48	6.96 ± 0.77	2.21 ± 0.04	8.85 ± 0.14
1	10.74 ± 0.92	10.74 ± 0.92	5.20 ± 0.56	8.32 ± 0.90	2.08 ± 0.07	8.32 ± 0.29
2	8.36 ± 1.14	8.36 ± 1.14	5.69 ± 0.53	9.11 ± 0.85	2.24 ± 0.13	8.97 ± 0.52
3	7.67 ± 0.12	7.67 ± 0.12	7.06 ± 0.14	11.29 ± 0.22	2.77 ± 0.05	11.06 ± 0.18
4	5.71 ± 0.70	5.71 ± 0.70	4.99 ± 0.31	7.98 ± 0.49	2.26 ± 0.07	9.03 ± 0.29
5	4.33 ± 0.35	4.33 ± 0.35	6.71 ± 1.57	10.74 ± 2.51	1.60 ± 0.07	6.39 ± 0.26
6	3.88 ± 0.21	3.88 ± 0.21	2.86 ± 0.65	4.57 ± 1.04	1.56 ± 0.04	6.22 ± 0.15
Stage 0–6	50.21 ± 2.61	50.21 ± 2.61	36.86 ± 1.43	58.97 ± 2.28	14.71 ± 0.42	58.84 ± 1.67
Stage 3–6	21.59 ± 1.70	21.59 ± 1.70	21.61 ± 1.93	34.58 ± 3.08	8.18 ± 0.58	32.7 ± 2.31
Stage 3–5	17.71 ± 1.68	17.71 ± 1.68	18.76 ± 1.11	30.01 ± 1.77	6.62 ± 0.59	26.48 ± 2.34

## Data Availability

The data that support the findings of this study are available from the corresponding author upon reasonable request.

## Abbreviations

ACI, Andersen cascade impactor; COPD, chronic obstructive pulmonary disease; DD, delivered dose; EDX, energy-dispersive X-ray; FF, fluticasone furoate; FPD, fine particle dose; FPF, fine particle fraction; GS, geometric standard deviation; HPLC, high-performance liquid chromatography; ICS, inhaled corticosteroid; LABA, long-acting beta-2 agonist; LAMA, long-acting muscarinic antagonist; MMAD, mass median aerodynamic diameter; NGI, Next Generation Impactor; SEM, scanning electron microscopy; VI, vilanterol trifenatate; UMEC, umeclidinium bromide.
